# Enhancing english oral translation through cross-modal learning and synchronous optimization

**DOI:** 10.1371/journal.pone.0329381

**Published:** 2025-08-18

**Authors:** Yan Wang

**Affiliations:** Anhui Wenda University of Information Engineering, Hefei, Anhui, China; Chuo University, JAPAN

## Abstract

Oral translation in English serves as a critical conduit for international communication and cultural exchange. However, the prevalent variations in pronunciation and the rapid pace of spoken language currently impede the efficacy of synchronous translation methods. To improve the quality and efficiency of synchronous oral translation, this paper explores the integration of cross-modal semantic understanding and synchronous enhancement specifically for English oral translation. This exploration commences with the implementation of a cross-modal translation scenario. Subsequently, the text sequence derived from this process is amalgamated with the original speech features via Bidirectional Encoder Representations from Transformers (BERT). The cross-information between modalities is explored, and linear transformation optimization is performed on the self-attention mechanism in Transformer to achieve context-awareness and understanding of oral-transcribed text. In conclusion, the integration of dynamic time warping (DTW) enhances real-time synchronization between speech and text, thereby improving translation fluency. Experimental results reveal that, when compared to the existing bilingual attention neural machine translation (NMT) model and the context-aware NMT model, the model proposed in this study yields an average bilingual evaluation understudy (BLEU) score that is 9.3% and 26.9% higher, respectively. Furthermore, its synchronization speed surpasses that of the other two models by 17.9% and 16.8%, respectively. These findings suggest that the fusion model, which incorporates context-awareness and an attention mechanism in cross-modal translation, can significantly elevate the quality and efficiency of English oral translation, offering a novel approach to the synchronous translation of spoken English.

## 1. Introduction

In the era of globalization, the importance of international communication has surged, particularly in realms such as international business, academic exchanges, and daily interactions, where the need for oral translation is increasingly evident [1,2]. However, contemporary translation algorithms often struggle to accurately interpret the contextual relationships and correlations among multiple modalities when handling multimodal information. As the field of information technology rapidly evolves, substantial advancements have been observed in natural language processing (NLP) [3]. The incorporation of context-awareness technology enhances the model’s ability to comprehend the overarching meaning of a sentence, while attention mechanisms bolster the model’s capacity to capture the sentence’s core information. In the domain of oral translation, integrating these elements and effectively leveraging multimodal information can lead to innovative cross-modal semantic understanding and simultaneous enhancement techniques. This holds significant theoretical and practical implications for fulfilling the growing demand for high-quality, real-time translation and advancing the practical utilization of intelligent translation technology.

This article explores the application of cross-modal semantic understanding and synchronous enhancement to English oral translation, with a view to enhancing both translation quality and efficiency, and meeting the demands of real-time translation. The study integrates context-awareness and attention mechanisms to achieve optimization in multimodal scenarios. When compared with models based on the bilingual attention neural machine translation (NMT) model and the context-aware neural machine translation (NMT) model, our model demonstrates superior performance in aspects such as translation accuracy, fluency, synchronization speed, and consistency. The average bilingual evaluation understudy (BLEU) score of our model surpasses that of the other two models by 9.3% and 26.9%, respectively. Further, our model shows an average perplexity that is 9.2% and 15% lower in relation to the other two types of models, respectively, and it also has a lower average grammar error rate. In terms of synchronization speed, our model’s average token processing speed per second exceeds that of the other two types of models by 17.9% and 16.8%, respectively. As for translation consistency, the mean translation edit rate (TER) of our model in each test subset reaches 15.6%, compared to the mean TER values of 18.9% and 20.2% for the bilingual attention NMT model and the context-aware NMT model, respectively. Our findings suggest that a fusion model based on context-awareness and attention mechanisms in cross-modal translation can enhance the real-time performance of English oral translation tasks while ensuring the quality of translation.

The research contributions of this article include:

(1) Cross-modal semantic understanding:

This paper presents a novel cross-modal semantic understanding mechanism that integrates visual and auditory data. This integration addresses the limitations of single-modal information in current models and mitigates the issues of reduced translation accuracy due to variations in accent and speech speed. Consequently, it enhances the comprehension of oral content in cross-modal contexts.

(2) Integration of context awareness and attention mechanisms:

The integration of context-aware techniques enhances the model’s ability to capture long-range dependencies, thereby augmenting its contextual sensitivity. The attention mechanism, refined through linear transformation, permits a more flexible allocation of attention weights. This flexibility enables superior capture of long-range dependencies within the input sequence, ultimately improving the translation quality of complex oral expressions.

(3) Synchronization enhancement

This article employs the Dynamic Time Warping (DTW) algorithm to facilitate real-time fusion of information during the processing of multimodal inputs, thereby synchronizing speech and text in real time. This approach enhances both the performance and accuracy of the translation. Furthermore, the model dynamically adjusts its dependence on various modal information when managing oral translation tasks.

## 2. Related work

Current English translation models may yield inaccuracies when handling rare vocabulary or intricate grammatical structures [4, 5]. To enhance translation efficacy, Khan S. N. integrated rule-based translation methods with artificial neural networks, creating an environment for efficient pattern matching and example learning. This integration led to the implementation of Neural Machine Translation (NMT), which achieved an n-gram BLEU score of 0.5903 across approximately 500 Hindi test sentences, thereby indicating superior translation quality [6]. Saini S. proposed a novel machine translation system, influenced by the sequential adaptive memory cognitive model. This system utilized a sequence-to-sequence learning method to comprehend and generate speech and language, reflecting the structure of the cerebral neocortex region. The experimental results confirmed that this method could deliver satisfactory, high-quality translation results [7]. In an effort to enhance grammatical accuracy in translation, Wang X. developed a fundamental English grammar analyzer for English-Chinese translation, improving upon the generalized maximum likelihood ratio algorithm. A character mapping function was employed to automatically recognize sentence boundaries, and same-level clauses were used to outline the grammatical structure of sentences. This facilitated the transition of proverbs from the original sentences to the target sentences. The effectiveness of this method was subsequently validated through example sentence analysis [8]. Koul N introduced an RNN (Recurrent Neural Network)-based NMT model to mitigate the challenges associated with translating long and atypical sentences. This recursive neural network was adeptly trained using sequence-to-sequence examples, thereby addressing uncommon scenarios such as extended sentences and rare lexical items. During the training phase, specific weighting factors were employed to account for the nuances of unusual words. Upon experimental evaluation, Koul N’s model demonstrated a notable 10% enhancement in translation accuracy when juxtaposed with a naive Bayes classifier [9]. While contemporary research has undeniably bolstered the precision of English translation, challenges persist, particularly in areas of single modality and inadequate contextual comprehension.

Cross-modal translation, which amalgamates information from diverse modalities, notably enhances translation accuracy [10,11]. By integrating context-awareness and leveraging multi-head attention mechanisms, one can more effectively delineate the dependency relationships among sentences [12,13]. To mitigate the gradient problem, Kang L introduced an NMT model predicated on bilingual attention, with experimental outcomes indicating its superior performance over mainstream Transformer models [14]. Premjith B employed long short-term memory (LSTM) and bidirectional RNN to construct an NMT system, adeptly addressing the challenge of translating extended sentences [15]. The context-based NMT algorithm proposed by Sharma V. K harnessed expansive unlabeled corpora and diminutive bilingual parallel corpora for translation, with experimental evaluations revealing its enhanced precision compared to baseline methods [16]. Ramadani R. A facilitated cross-modal translation by transmuting audio into text, processing it, and aligning it with a sign language system; tests affirmed a commendable level of accuracy [17]. While cross-modal translation augments context comprehension and translation quality by meticulously analyzing multi-modal data, it persistently endeavors to fulfill real-time requisites for synchronous translation across varied modalities. A majority of the extant cross-modal translation algorithms are deeply rooted in neural networks. Although they proficiently navigate everyday translation challenges, they grapple with thoroughly comprehending intricate syntactic and syntax connections, thereby impairing translation quality. Moreover, regarding the management of rare words, numerous models presently lack the capability to effectively augment the dictionary, leading to mistranslations or omissions.

## 3. English oral translation with the integration of context-awareness and attention mechanism

### 3.1. Multimodal data integration

**Fig 1:**
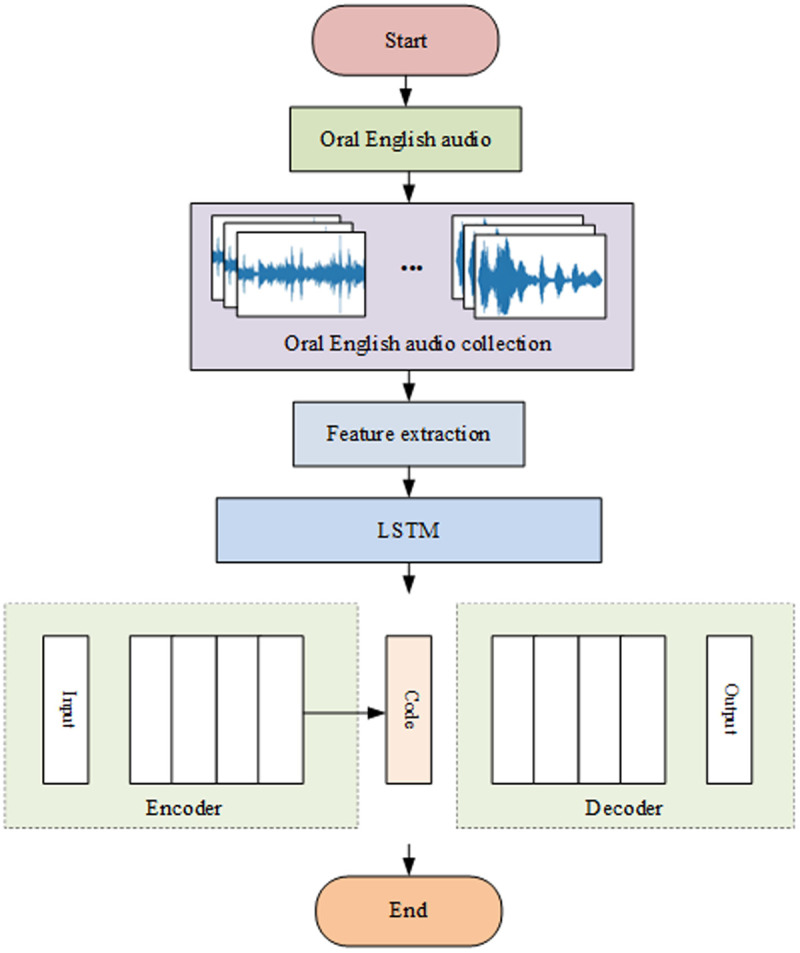
Speech recognition process.

Multimodal data fusion is a process that amalgamates data from diverse sources and formats for all-encompassing analysis [18]. This paper focuses on the integration of speech and text data. The initial step involves utilizing Long Short-Term Memory (LSTM) to scrutinize the audio features of spoken English and to recognize the corresponding oral content. Subsequently, these recognized results are depicted as probability distributions, paving the way for modal fusion. Upon decoding the speech, the textual information is transformed into coherent text data, a process illustrated in Fig 1.:

The collected oral English audio sequence is represented as $\text{X}=\left\{{\text{x}}_{\text{1}},{\text{x}}_{\text{2}},\cdots ,{\text{x}}_{\text{T}}\right\}$, where T is the length of the sequence. Mel frequency cepstrum coefficients are used for feature extraction to obtain feature sequence $\text{S}=\left[{\text{s}}_{\text{1}},{\text{s}}_{\text{2}},\cdots ,{\text{s}}_{\text{m}}\right]$. The expression t of the input gate, forget gate, and output gate for each time step is processed with $\text{S}=\left[{\text{s}}_{\text{1}},{\text{s}}_{\text{2}},\cdots ,{\text{s}}_{\text{m}}\right]$ as input. The formulas are [19,20]:


$$i_{t}=\sigma (W_{xi}x_{t}+W_{hi}h_{t-1}+b_{i})$$ (1)

$$f_{t}=\sigma (W_{xf}x_{t}+W_{hf}h_{t-1}+b_{f})$$ (2)

$$o_{t}=\sigma (W_{xo}x_{t}+W_{ho}h_{t-1}+b_{o})$$ (3)

Table 1 presents the definitions of the variables in Formulas (1) to (3):

**Table 1:** Definitions of the variables in Formulas (1) to (3).

Classification	Variables	Meaning
Input gate	$i_{t}$	Output
	$W_{xi}$	Weight matrix from input to input gate
	$W_{hi}$	Weight matrix from hidden state to input gate
	$b_{i}$	Bias vector
Forget gate	$f_{t}$	Output
	$W_{xf}$	Weight matrix from input to forget gate
	$W_{hf}$	Weight matrix from hidden state to forget gate
	$b_{f}$	Bias vector
Output gate	$o_{t}$	Output
	$W_{xo}$	Forgetting matrix from input to output gate
	$W_{ho}$	Weight matrix from hidden state to output gate
	$b_{o}$	Bias vector

Using the LSTM gating mechanism, the mechanism and hidden state ${\text{h}}_{\text{t}}$ at the current time can be calculated through the formulas [21]:


$${\tilde{c}}_{t}=tanh(W_{xc}x_{t}+W_{hc}h_{t-1}+b_{c})$$ (4)

$$c_{t}=f_{t}⊙c_{t-1}+i_{t}⊙{\tilde{c}}_{t}$$ (5)

$$h_{t}=o_{t}⊙tanh(c_{t})$$ (6)

Table 2 presents the definitions of the variables in Formulas (4) to (6):

**Table 2:** Definitions of the variables in Formulas (4) to (6).

Sequence	Variables	Meaning
1	${\tilde{c}}_{t}$	Candidate cell states
2	⊙	Element-wise multiplication
3	$W_{xc}$	Inputting the weight matrix to ${\tilde{c}}_{t}$
4	$x_{t}$	Audio sequence at time step
5	$W_{hc}$	Hiding the weight matrix of the state to ${\tilde{c}}_{t}$
6	$b_{c}$	Bias vector of ${\tilde{c}}_{t}$
7	$c_{t-1}$	Cell state of time step

Audio files undergo preprocessing, after which their features are extracted. These features are then fed into the LSTM network, where speech processing is executed based on specific gating mechanisms. The use of LSTM facilitates the transfer of feature information to the fully connected layer, endowing the classification output with the requisite nonlinear transformation capability. Subsequently, the probability distribution of characters that might occur at each time point is predicted by transforming the results of the fully connected layer via Softmax. This process enables the joint temporal classification loss function to decode the probability distribution of characters and generate a series of text sequences.

The English oral translation system is enhanced by integrating visual information, specifically lip movements, as an additional input modality alongside speech and text data. Utilizing lip-reading technology, both speech and text inputs can be processed. This allows for the use of visual information to decode alterations in mouth shape, thereby providing supplementary clues particularly beneficial in instances where the speech signal is unclear or disrupted by noise interference.

**Fig 2:**
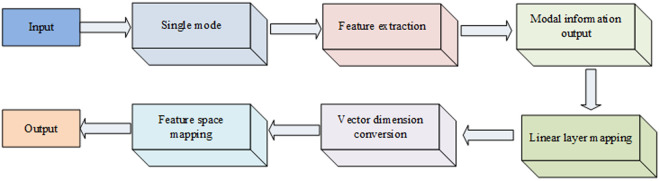
Multimodal fusion.

BERT, a pre-trained language model, possesses a robust capability for processing and understanding natural language texts [22,23]. In this study, BERT is employed to integrate the derived text sequence with the original speech features, thereby creating a unified modal representation. To more effectively harness the cross-modal information and the long-term relationships within each modality, a joint feature representation is adopted for the vectors. This process encompasses embedding data from three modalities and amalgamating multiple vectors from a singular modality to produce a comprehensive multimodal vector, as depicted in Fig 2.

In Fig 2, it is postulated that corresponding features are extracted from N modal data and subsequently transformed via a feature extraction network. The single-mode features of text, sound, and visual information modes are mapped onto a linear layer, facilitating the high-dimensional conversion of these three single-mode features; this entails converting the output vector dimension to a higher one. Ultimately, the feature vectors from both modalities are projected into the global feature space [24,25]. In Fig 2, N linear layers, corresponding to the three modal vectors, are employed to transform N feature vectors.

**Fig 3:**
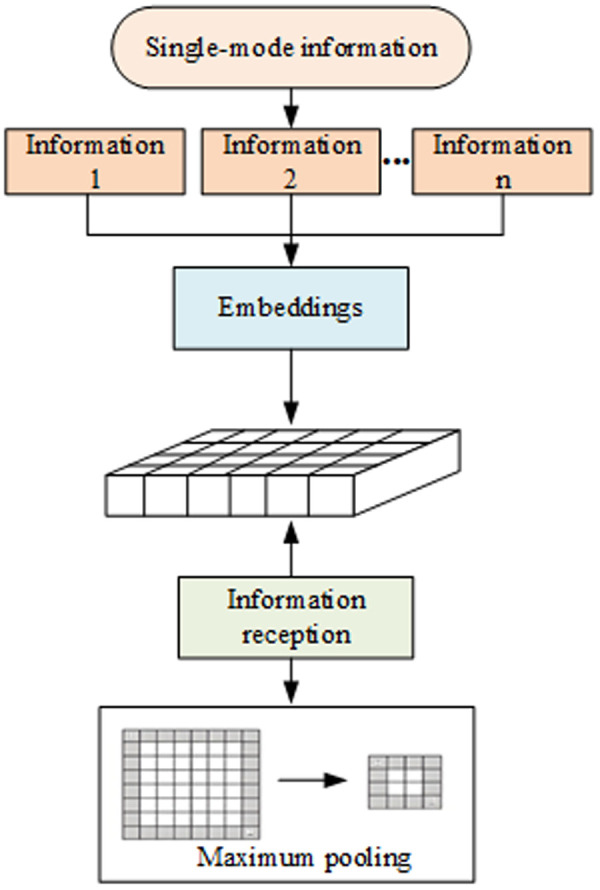
Maximum pooling operation.

In order to ensure a distinct embedding for each modality, the BERT vector embedding simulates the process by embedding all the information from each individual modality, symbolized by. During the fusion of feature vectors, all information pertaining to the modality is accepted, and the features of that modality are pooled to the maximum extent. This process is illustrated in Fig 3:

According to Fig 3, after performing the maximum pooling operation, the most significant feature in this modality is taken as the initial value for the fused feature:


$$E_{Agg}=maxpool\left({\left\{E\right\}}_{\tau =1}^{\tau }\right)$$ (7)

The sequence representation of input features fused with feature vectors is:


$$E_{Token}=\left[E^{1},E^{1},\cdots ,E^{1},\cdots ,E^{N},E^{N},\cdots ,E^{N}\right]$$ (8)

In order to process multimodal data efficiently, the positions of each modality are encoded separately using a modal embedding, and corresponding models are subsequently established. During the process of positional embedding, a modal encoding method is employed to ensure that the input feature sequence is distinguishable among different modalities. For N modal data, each modality is recognized by the modal code of N positions, and the coding dimension of each modal position is ${\text{d}}_{\text{model}}$. The position modal encoding sequence of the fused vector is represented as:


$$E_{position}=\left[E_{agg}^{1},E_{1}^{1},\cdots ,E_{\tau_{1}}^{1},\cdots ,E_{agg}^{N},E_{1}^{N},\cdots ,E_{\tau_{N}}^{N}\right]$$ (9)

The feature vectors of each modality contain semantic and modal information. On this basis, a unified modal representation ${\text{M}}_{\text{total}}$ is defined by the formula:


$$M_{total}=E_{Token}+E_{position}=\left[\omega_{agg}^{1},\omega_{1}^{1},\cdots ,\omega_{\tau_{1}}^{1},\cdots ,\omega_{agg}^{N},\omega_{1}^{N},\cdots ,\omega_{\tau_{N}}^{N}\right]$$ (10)

### 3.2. Construction of context-awareness model and optimization of attention mechanism

The Transformer model boasts flexibility and scalability in the field of Natural Language Processing (NLP) [26,27]. The resulting unified modal expression ${\text{M}}_{\text{total}}$ is input into the Transformer network. On this basis, the self-attention mechanism in Transformer is utilized to perform context-awareness and understanding of oral-transcribed text. By establishing a contextual model to determine each part of the text sequence, omissions and non-standard grammar can be recognized.

**Fig 4:**
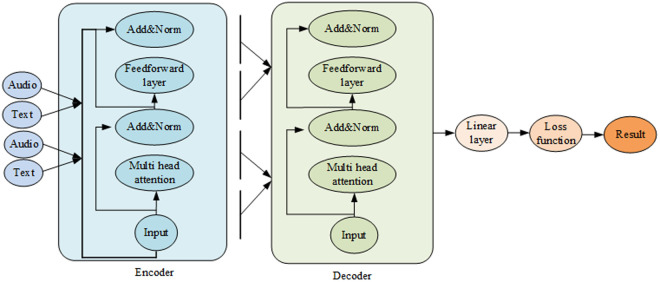
Context-awareness model.

The context-awareness model is predicated on the architecture of the Transformer encoder, illustrated in Fig 4.

In Fig 4, the Add&Norm function incorporates residual connections and Layer-Normalization operations. The role of residual connections is to mitigate the loss of gradient during the backpropagation process. On the other hand, Layer-Normalization serves as a normalized method capable of discerning feature differences within a singular sample, thereby enhancing the stability of the model. In the context of feedforward neural networks, fully connected layers and ReLU activation functions are employed.

The Transformer encoder’s primary role is to convert the input into a hidden layer, a mathematical representation encompassing natural language sequences [28]. The decoder subsequently maps this hidden layer back to the natural language sequence, thus allowing the semantic model to address complex problems effectively [29,30]. In accordance with Bayes’ rule, given the context encoding sequence and the initial target vectors for each target variable, the distribution is broken down into the product of the conditional distributions for each target vector:


$$\begin{array}{c} p_{\theta_{dec}}(Y_{1:m}|X_{1:n}\;=\prod {\mathrm{\backslash nolimits}}_{i=1}^{m}p_{\theta_{dec}}\left(y_{i}|Y_{0:i-1},X_{1:n}\right)) \end{array}$$ (11)

The limitations of self-attention mechanisms in creating effective models for high-dimensional input vectors necessitate the establishment of multi-head attention mechanisms [31,32]. ${\text{Q}}_{\text{1}},\;{\text{K}}_{\text{1}},\;{\text{V}}_{\text{1}}$ are replaced with matrix coefficients ${\text{W}}_{\text{1}}^{\text{Q}},\;{\text{W}}_{\text{1}}^{\text{K}},\;{\text{W}}_{\text{1}}^{\text{V}}$, and they are mapped to a semantic space 1, as shown in Formula (12):


$$Q_{1}=QW_{1}^{Q}=[\begin{array}{c} q_{1}W_{1}^{Q}\\ \cdots \\ q_{m}W_{1}^{Q} \end{array}],K_{1}=KW_{1}^{K}=[\begin{array}{c} q_{1}W_{1}^{K}\\ \cdots \\ q_{m}W_{1}^{K} \end{array}],V_{1}=KW_{1}^{V}=[\begin{array}{c} q_{1}W_{1}^{V}\\ \cdots \\ q_{m}W_{1}^{V} \end{array}]$$ (12)

Here, Q, K, V represent input features. On this basis, the Attention operation is performed and expressed as [33]:


$$Z={head}_{1}=Attention(Q_{1},K_{1},V_{1})$$ (13)

${\text{head}}_{\text{1}}$ represents a single vector sequence, and its length is the same as Q. By using matrix coefficients ${\text{W}}_{\text{1}}^{\text{Q}},\;{\text{W}}_{\text{1}}^{\text{K}},\;{\text{W}}_{\text{1}}^{\text{V}}$ to transform input features Q, K, V into semantic space 2, the Attention operation is repeatedly performed to obtain ${\text{head}}_{\text{2}}$. Similarly, ${\text{head}}_{\text{3}}\cdots {\text{head}}_{\text{c}}$ can also be obtained.

Finally, by integrating the formula, a complete multi-head attention representation is obtained:



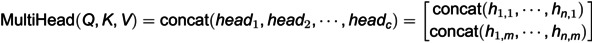

(14)


**Fig 5:**
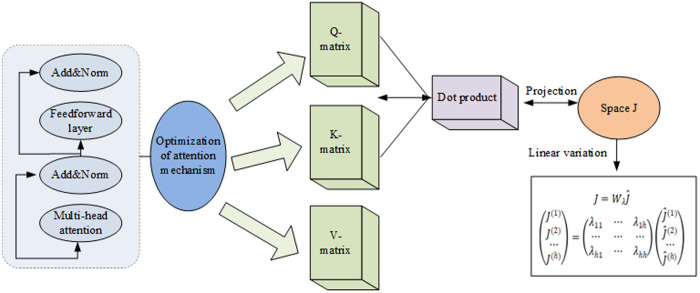
Optimization of attention mechanism.

In multimodal scenarios, each modality’s data is subjected to numerous Attention operations, potentially impacting the model’s learning efficiency and effectiveness. This paper employs a linear transformation method to compute multiple head attention points and aggregates them, thereby enhancing the efficiency and capacity of attention representation. This approach is depicted in Fig 5.

The linear transformation incorporates an additional linear layer into the traditional attention computation, thereby enhancing attention efficiency. During the calculation of queries, keywords, and values, this paper employs a weighted matrix for linear transformation alongside the original word embedding vectors. This approach augments the model’s capacity to capture input features, rendering the attention mechanism more versatile in weight distribution and more effective in identifying long-distance correlations within sequences. Through strategic adjustments to the weight matrix in linear transformation, an adaptive modification of attention allocation strategies under varying input conditions can be accomplished. Consequently, the representation ability and generalization effect of the model are significantly improved.

Each modality’s input data undergoes a linear transformation to generate various attention heads, which are subsequently weighted together for an integrated expression. This approach to multi-attribute recognition, based on sparse representation, effectively addresses the prevalent issue of redundancy in traditional multi-attribute recognition algorithms. It also minimizes unnecessary calculations, thereby enhancing the efficiency of multimodal data processing.

The output vector is ultimately derived by merging and transforming the results of each attention head through an additional linear transformation matrix. This “multi-head” architecture not only bolsters the capture of intricate patterns but also augments computational efficiency via parallel processing. Building on this, the paper employs hierarchical normalization and residual connectivity techniques to ensure robust learning and performance enhancement of self-interested factors. Consequently, the model can concentrate more effectively on pertinent information without being disturbed by inter-modal noise.

Firstly, by obtaining Q, K, V, the dot product of Q and K below the i-th head is calculated and represented as $\hat{\text{J}}$. The calculation method is shown in Formula (15) [34,35]:


$${\hat{J}}^{\left(1\right)}=Q^{\left(1\right)}{\left(K^{\left(1\right)}\right)}^{T\;}$$ (15)

The parameter matrix is multiplied with the calculation result so that each dot product contains the attention information of all heads, and it is projected to space J. ${\text{W}}_{\lambda }$ is used to represent the parameter matrix. The linear transformation is calculated in Formula (16):


$$\left\{ \;\;\;\;\begin{array}{c} J=W_{\lambda }\hat{J}\\ \left(\begin{array}{c} J^{\left(1\right)}\\ J^{\left(2\right)}\\ \begin{array}{c} \cdots \\ J^{\left(h\right)} \end{array} \end{array})=(\begin{array}{ccc} \lambda_{11} & \cdots & \lambda_{1h}\\ \cdots & \cdots & \cdots \\ \lambda_{h1} & \cdots & \lambda_{hh} \end{array})(\begin{array}{c} {\hat{J}}^{\left(1\right)}\\ {\hat{J}}^{\left(2\right)}\\ \begin{array}{c} \cdots \\ {\hat{J}}^{\left(h\right)} \end{array} \end{array}\right) \end{array}\right.\;$$ (16)

In Formula (16), λ represents a parameter in a matrix. Its spatial information is normalized using Softmax and then multiplied by a numerical matrix to obtain the head attention. All the head attentions are concentrated and multiplied by matrix ${\text{W}}^{\text{o}}$ to obtain mixed multi-attention. Formula (17) shows the calculation process:


$$\left\{ \;\;\;\begin{array}{c} P^{\left(i\right)}==softmax(J^{\left(i\right)})\\ O^{\left(i\right)}=P^{\left(i\right)}V^{\left(i\right)}\\ O=\left[O^{\left(1\right)},O^{\left(2\right)},\cdots ,O^{\left(i\right)},\cdots ,O^{\left(h\right)}\right]{\text{W}}^{\text{o}} \end{array}\right.\;$$ (17)

In Formula (17), ${\text{P}}^{\left(\text{i}\right)}$ represents the value after spatial normalization; ${\text{V}}^{\left(\text{i}\right)}$ represents the value matrix; ${\text{O}}^{\left(\text{i}\right)}$ represents the head attention. Mixed head attention can correlate information from multiple heads of attention, thereby improving the translation quality of the model.

### 3.3 Synchronous enhancement algorithm

Dynamic Time Warping (DTW) serves as a metric for gauging the similarity between two time series, allowing them to be matched [36]. The DTW algorithm is adept at identifying the optimal alignment path between two sequences, even in instances where their timing isn’t strictly synchronized. It essentially seeks a pathway between two sequences that minimizes the cumulative distance of the entire route [37]. In a cross-modal translation context, there often exists a pronounced temporal mismatch between speech and text. This discrepancy arises as speakers’ paces can cause the content of a speech to either extend or contract in duration. Conventional timing window alignment techniques prove versatile in addressing such inherent timing inconsistencies. However, the DTW algorithm stands out by enabling precise positioning, owing to its ability to search for the best possible path based on temporal shifts. This method formulates a two-dimensional cost matrix where each entry denotes the distance between corresponding positions of the two sequences. Leveraging dynamic programming principles, it then discerns the most abbreviated cumulative route from its inception to its ultimate destination. Employing the DTW algorithm for data alignment ensures meticulous mapping and transformation of information across different modalities during conversion. Moreover, the robust adaptability of the DTW algorithm renders it well-suited for managing both linear temporal relationships and intricate temporal structures inherent in multimodal data within cross-modal translation tasks.

**Fig 6:**
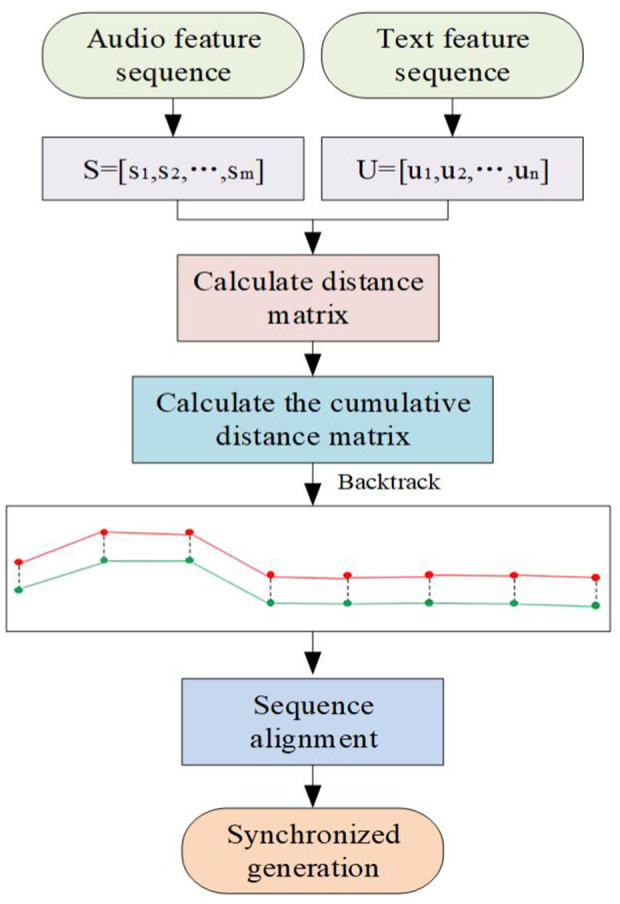
Sequence synchronous enhancement.

This article addresses the issue of processing time series with varying lengths by utilizing the Dynamic Time Warping (DTW) algorithm for time series alignment. This is achieved through the development of a context-aware mechanism and the optimization of an attention mechanism, which facilitate real-time synchronization between speech and text. Consequently, the fluency of translation is enhanced. The process is illustrated in Fig 6.

Assuming that the oral audio feature sequence is $\text{S}=\left[{\text{s}}_{\text{1}},{\text{s}}_{\text{2}},\cdots ,{\text{s}}_{\text{m}}\right]$ and the text feature sequence is $\text{U}=\left[{\text{u}}_{\text{1}},{\text{u}}_{\text{2}},\cdots ,{\text{u}}_{\text{n}}\right]$, Euclidean distance is used to calculate the distance matrix D between S and U:


$$D[i,j]={\left\|s_{i}-u_{j}\right\|}^{2}$$ (18)

Cumulative distance matrix A is calculated:


$$A[i,j]=\left\{ \;\;\;\begin{array}{c} D[i,j],\;if\;i=0\;or\;j=0\\ D[i,j]+min\left(A[i-1,j],A[i,j-1],A[i-1,j-1]\right),\;otherwise \end{array}\right.\;$$ (19)

Starting from the bottom right corner A[m,n] of matrix A, the optimal path P is found through backtracking:


P={(i,j)} (20)

The principle of backtracking is to move along the direction of minimum value:


$$path(i,j)=\left\{ \;\;\;\begin{array}{c} \left(i-1,j),\;if\;A[i-1,j]\leq A[i,j-1and\;A[i-1,j]\leq A[i-1,j-1\right]\\ \left(i,j-1),if\;A[i,j-1]\leq A[i,j-1]\;and\;A[i,j-1]\leq A[i-1,j-1\right]\\ (i-1,j-1),\;otherwise \end{array}\right.\;$$ (21)

The conventional Dynamic Time Warping (DTW) method exhibits reduced computational efficiency when applied to data with extended time series. This article uses Locality Sensitive Hashing (LSH) to preprocess speech sequences and maps them to a low dimensional space to improve matching efficiency. Firstly, using the LSH algorithm, its dimensionality is reduced, and H(X) is defined as a set of hash functions. In the low dimensional space, the audio sequence data X is expressed as:


$$X^{‘}=\left[h_{1}(X),h_{2}(X),\cdots ,h_{k}(X)\right]$$ (22)

On this basis, feature extraction is performed on the data processed by LSH to capture temporal dependencies in the sequence. The oral audio feature sequence S is matched with the text feature sequence U. The aligned sequence φ is:


$$\varphi =\left[\varphi_{1},\varphi_{2},\cdots ,\varphi_{k}\right]$$ (23)

The Dynamic Time Warping (DTW) algorithm’s matching results are seamlessly integrated with the module. We apply a context-aware model, which is combined with an optimized linear variation-based attention mechanism, to influence the final translation decision process collectively. The translation is executed via an aligned sequence, facilitating the automatic generation of the target language text.

## 4. English oral translation experiment

### 4.1 Experimental data

**Fig 7:**
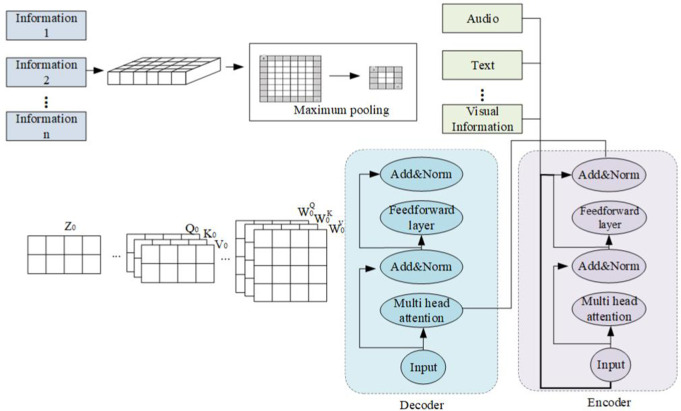
Translation model in this article.

The translation model in this article is shown in Fig 7.

This study utilizes LibriSpeech as the experimental dataset, which provides both text and audio formats. It comprises approximately 1000 hours of 16kHz read English speeches, meticulously segmented and normalized. These speeches are divided into audio files of roughly 10 seconds each, accompanied by text annotations. Prior to experimental analysis, both audio and text data from the original dataset undergo preprocessing. The audio files are edited to remove noisy sections, while the text data is stripped of special characters and punctuation, retaining only standard text. Text differentiation is achieved through the use of lowercase letters, and it is further segmented into sub-word units to create a vocabulary.

To simulate a diverse spoken language environment and ensure model adaptability to dialects and intricate structures, additional processing is performed on the experimental dataset. Extra oral English data is sourced from the TED talk dataset, with the supplementary corpus featuring distinctly marked Indian English, Australian English, Irish English, Scottish English, and Southern English. Conversational data from the Common Voice database is also included. To reflect real-world usage scenarios, the Switchboard corpus provides additional voice data from everyday phone conversations. Texts from the Penn Treebank database and COCA Complex Sentences that contain nested clauses, split sentences, and inverted sentences undergo speech conversion, representing 12% of the corpus. Moreover, the CELEX corpus is employed to embed specialized terms for high-frequency applications into various texts, such as news reports, academic speeches, and classroom discussions, thereby generating new speech samples. The refined dataset comprises 1200 hours of speech samples, with the dataset feature breakdown detailed in Table 3.

**Table 3:** Summary Table of Key Dataset Characteristics.

Dataset Name	Size (Hours)	Source Description	Variability Features	Data Format
LibriSpeech	~1000	LibriVox project recordings of read English speeches	Multiple speakers, dialects, and topics	10-second audio clips with text annotations
TED Talk Dataset	~120	TED talks covering various subjects with speakers from different countries	Indian English, Australian English, Irish English, etc.	Talks with aligned transcripts
Common Voice Database	~40	Mozilla’s Common Voice project with Scottish and Southern English conversations	Conversational Scottish and Southern English	Speech with transcripts
Switchboard Corpus	~300	Recorded telephone conversations between speakers in the United States	Everyday spoken English in conversational contexts	Transcribed conversations segmented into units
Penn Treebank & COCA Complex Sentences	~60	Penn Treebank and COCA databases with complex grammatical structures	Embedded clauses, split sentences, inverted sentences, etc.	Speech converted from complex texts with original text alignment
CELEX Corpus	~80	CELEX corpus with high-frequency specialized terms	Specialized vocabulary from news, academia, classrooms, etc.	Speech samples with integrated specialized terms and annotations

The combination of these datasets provides a large and diverse data source, which is beneficial for the training of the English oral translation model. LibriSpeech dataset is the basic part, and other datasets make up the diversity of dialects, conversational style, complex grammar and special vocabulary. The use of this large-scale dataset has made the translation model more accurate, fluent and robust in different scenarios of oral translation.

To mitigate potential translation bias and safeguard speech data privacy, this study employs the Word Embedding Association Test to ascertain the presence of gender, race, and other biased vocabulary in training samples. By detecting such hidden biases, techniques like semantic substitution and data class balancing are implemented to diminish model output bias. Furthermore, the voice anonymity algorithm, predicated on Voice Masking, prevents the unauthorized disclosure of users’ voice identities. This is achieved by ensuring non-identification of voice data while maintaining voice recognition. Moreover, differential confidentiality approaches are utilized to secure data.

The oral data collected is automatically segmented into approximately 10-second intervals using the Librosa library in Python. These segmented audio clips are then annotated with text using the original audio paired with subtitles. For consistency with the LibriSpeech data, all audio files are converted to the WAV format. This processed oral data is subsequently integrated with the LibriSpeech dataset, resulting in a comprehensive corpus encompassing both formal reading and oral expressions. The entire corpus is categorized into 10 subsets based on specific spoken language scenarios. The composition of the final experimental data is detailed in Table 4.

**Table 4:** Basic information of experimental data.

Sequence	Files	Duration (h)	Vocabulary
Test-01	5163	15.2	81421
Test-02	5012	14.9	80344
Test-03	4968	14.8	79627
Test-04	5231	15.5	86774
Test-05	5157	15.2	83019
Test-06	5044	14.9	87452
Test-07	5179	15.3	81794
Test-08	4822	14.5	80329
Test-09	5135	15.5	83372
Test-10	5279	15.8	90364

To assess the efficacy of the model presented in this paper, we compare it with existing cross-modal translation research using experimental data from Table 4. Specifically, we evaluate its performance against two baseline models: the bilingual attention-based NMT model from reference 14 and the context-aware NMT model from reference 16. The bilingual attention-based NMT model employs an encoder-decoder architecture that dynamically adjusts focus during cross-modal translation by computing attention weights between the source and target languages. In contrast, the context-aware NMT model integrates multimodal information to form context representations, leveraging context windows and guiding generation during decoding through context vectors.

In our experiments, we utilize a rigorous experimental setup to guarantee the reliability and validity of the model. For training, we meticulously curated a comprehensive dataset encompassing LibriSpeech, TED talk dataset, Common Voice database, Switchboard corpus, Penn Treebank database, COCA Complex Sentences, and CELEX corpus. Each dataset underwent preprocessing to eliminate noise and extraneous data, and the audio data was partitioned into roughly 10-second segments, accompanied by text annotations. Model parameters were initialized using the Xavier initialization method to preserve the variance of the activations and gradients. Training was executed using an Adam optimizer with a learning rate of 0.001. We conducted 50 training sessions with a batch size of 32, shuffling the training data at each session to maintain diversity. The model’s learning was guided by a combination of cross-entropy loss and attentional loss. Post-initial training, we fine-tuned the model via domain adaptation, employing domain-specific subsets, data enhancement techniques, and iterative improvements involving multiple training and evaluation cycles. Experiments were performed on a high-performance computing cluster featuring NVIDIA® 3090 GPUs, multi-core CPUs, and 512GB of RAM. The software environment comprised the PyTorch deep learning framework and various Python libraries such as NumPy, SciPy, pandas, Librosa, NLTK, and SpaCy for data and text processing tasks. The parameter settings of the model in this study are detailed in Table 5.

**Table 5:** Model parameter settings.

Category	Parameter	Parameter values
Architecture parameters	Hidden layer size	256
	Number of layers	2
	Attention head count	8
Training parameters	Learning rate	0.001
	Batch size	32
	Epoch	50

In the experiment, we tuned the main hyperparameters to achieve the best model performance. The learning rate and batch size are selected by grid and random search within a specific range of values. Specifically, the learning rate is searched between 0.0001 and 0.01, and finally 0.001 is chosen as a balance between convergence speed and model stability. Similarly, the batch size is searched between 16 and 64, and 32 is chosen as a balance between memory consumption and training efficiency. Meanwhile, we observed the model performance on the validation set to select the hyperparameters that produced the lowest validation loss and the highest translation accuracy.According to the settings of the model parameters, the model is trained and tested in each sub-set. We use the same parameter settings for bilingual attention NMT model and context-aware NMT model. As shown in the experimental results, the model converges after 50 training epochs and the BLEU score increases gradually before it converges to a stable value. Moreover, the proposed model has a lower error rate and syntax error rate, which indicates that the proposed model has a good translation fluency. The synchronization speed and translation consistency of the proposed model are also better than those of the baseline model.

In this study, we strictly adhered to ethical guidelines for data use. All experimental data were sourced from publicly available academic databases, such as LibriSpeech and the TED Talk Dataset, and their use complied with the licensing agreements of the data providers. Additionally, to protect user privacy, we employed speech anonymization algorithms to ensure that speech data could not be identified as belonging to specific individuals. Furthermore, we conducted a Word Embedding Association Test to identify and address any potential biased vocabulary in the training data, thereby reducing bias in the model’s outputs. All these measures were implemented to ensure the ethical integrity of the research and the legitimacy of the data.

### 4.2. Evaluation indicators

(1) Translation accuracy

The BLEU score is employed to evaluate the accuracy of translation, using the similarity between the generated translation results and the reference translation as the primary evaluation criterion. A high BLEU score suggests a substantial agreement between the model’s interpretation effect and the reference translation [38,39]. The calculation formula is as follows:


$$\begin{array}{c} BLEU=BP\cdot \left(\sum {\mathrm{\backslash nolimits}}_{n=1}^{N}w_{n}logp_{n}\right) \end{array}$$ (24)

Among them, there is:


$$BP=\left\{ \;\;\;\begin{array}{c} 1,\;if\;c>r\\ e^{1-\frac{r}{c}},\;if\;c\leq r \end{array}\right.\;$$ (25)

In Formulas (24) and (25), BP is the length penalty factor; $w_{n}$ and $p_{n}$ represent the weight and precision of the n-gram, respectively; c is the length of the candidate translation; r is the closest translation length to c in the reference translation.

(2) Translation fluency

The fluency of translation is assessed using two metrics: perplexity and grammar error rate. Perplexity, in particular, serves as an indicator of the fluency and naturalness of the text [40]. On the other hand, the grammar error rate quantifies the ratio of grammatical errors to the total word count. The formula for computing perplexity is as follows:


$$\begin{array}{c} Perplexity=exp\left(-\frac{1}{N}\sum {\mathrm{\backslash nolimits}}_{t\in T}logP(t)\right) \end{array}$$ (26)

N represents the total number of words in the sentence, and P(t) represents the logarithmic probability of the sentence.

(3) Synchronization speed

The synchronization speed is quantified using the metric “tokens per second,” which denotes the number of tokens processed each second. A higher value for this indicator corresponds to a more rapid synchronization speed in oral translation.

(4) Translation consistency

Translation consistency is quantified using TER, which assesses the editing distance between the translation output of each model and the corresponding reference translation. This metric indicates the frequency of required edits in relation to the total number of words in the reference translation.

(5) METEOR

Compared to BLEU, METEOR focuses more on the semantic similarity between the translation and the reference translation, rather than strict lexical matching. It can better reflect the quality of the translation, especially in terms of grammar changes, vocabulary usage, and other aspects. Its calculation formula is as follows:


$$METEOR=\frac{10\times Precision\times Recall}{\left(Precision+Recall)+\alpha \times (1-F\right)}$$ (27)

### 4.3. Experimental results

(1) Comparison of translation accuracy

**Fig 8:**
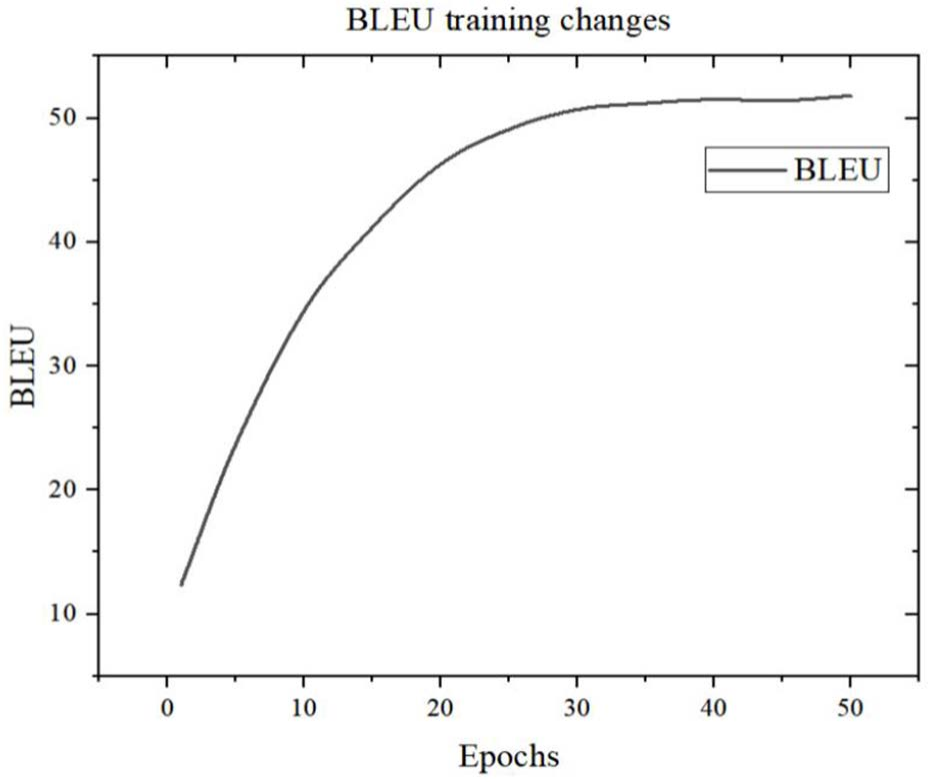
BLEU training changes.

The variation of BLEU model under the number of training rounds in this article is shown in Fig 8.

As illustrated in Fig 8, the BLEU score experiences a rapid ascent from 12.4 to 41.2 during the initial phases of training, thereby indicating the model’s swift adaptation to fundamental translation patterns. This early progress can largely be attributed to the model’s capacity for identifying prevalent language patterns from its original random state and for learning relatively straightforward syntactic and lexical alignment relationships. However, a deceleration in the growth of the BLEU score is evident from the 16th epoch onwards, with the rate of improvement gradually diminishing. This suggests that the model incrementally masters basic translation rules and starts addressing more intricate syntactic structures and semantic relationships. Overall, the model exhibits high training efficiency, achieving performance convergence within a confined number of training iterations.

**Fig 9:**
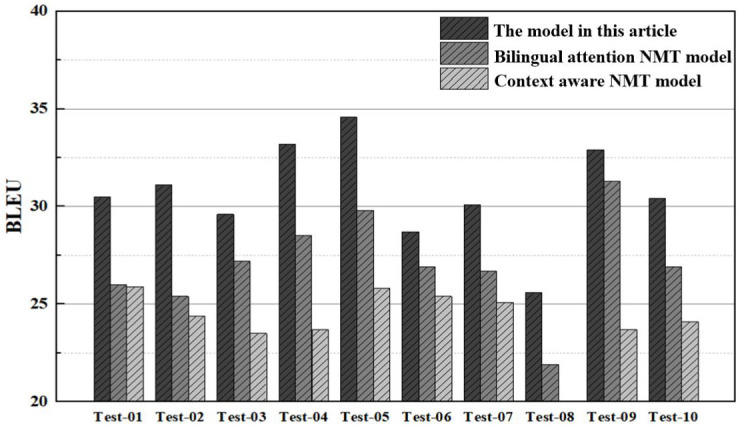
Comparison results of BLEU.

The BLEU scores of the model in this article are compared with the bilingual attention NMT model and the context-aware NMT model in each test subset, and its ability to handle complex language structures and long-term dependencies is explored. The results are shown in Fig 9.

As illustrated in Fig 9, the model presented in this article outperforms both the bilingual attention Neural Machine Translation (NMT) model and the context-aware NMT model across all test subsets in terms of BLEU score. Specifically, the average BLEU score for the model in this article is approximately 30.7, compared to 28.1 and 24.2 for the other two models, respectively. This represents an improvement of about 9.3% and 26.9% over these models, respectively. The bilingual attention NMT model struggles with effective integration of multimodal information, leading to issues such as incomplete oral translation. Similarly, the context-aware NMT model tends to overlook details during long sequence translations. In contrast, the model in this article harmoniously merges speech and text data, thereby leveraging a broad spectrum of information resources and enhancing translation comprehension for each test set, ultimately resulting in more accurate translations.

(2) Comparison of translation fluency

**Fig 10:**
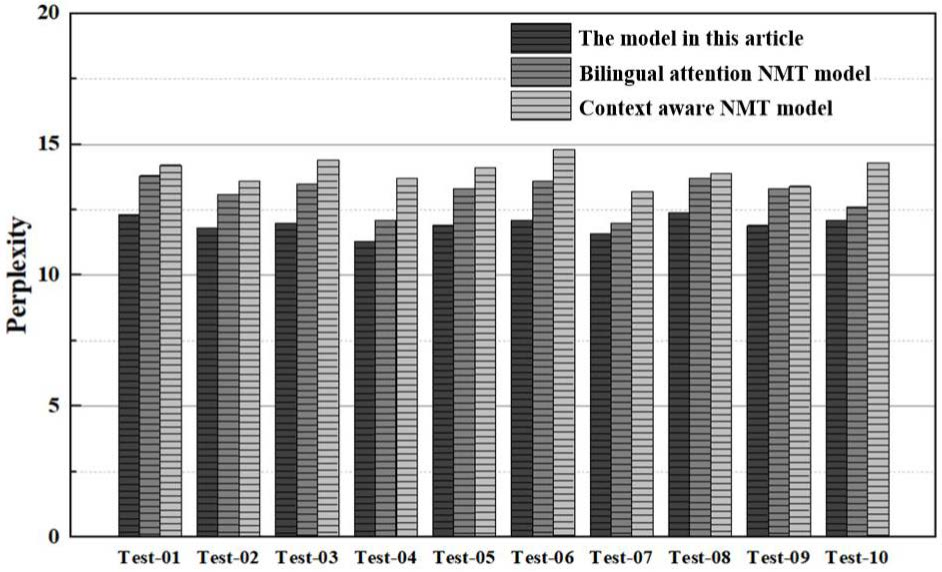
Comparison results of perplexity.

In the analysis of translation fluency, a comparison of the perplexity results and grammar error rate results of various models is made, as shown in Fig 10 and Table 5.

The perplexity comparison illustrated in Fig 10 demonstrates that the model articulated in this article consistently outperforms its counterparts across all subsets, exhibiting notably lower perplexity results. A comparative analysis of the average perplexity reveals that the model discussed in this paper yields an average perplexity of approximately 11.9, whereas the translation models founded on bilingual attention NMT and context-aware NMT yield average perplexities of 13.1 and 14.0, respectively. This indicates that the model in this article is superior by margins of 9.2% and 15%, respectively. The attention mechanism has been optimized through linear variation within the article, thereby focusing attention across various text segments and assigning appropriate weights during translation to enhance text coherence. The relatively limited capacity of both the bilingual attention and context-aware NMT models to extrapolate distant information could potentially compromise the coherence and fluency of the translation.

In Table 6, the model presented in this article boasts an average grammar error rate of 2.5%, compared to the averages of 3.5% and 3.9% for the other two models. These comparative results indicate that the grammar accuracy of the translations produced by our model is superior. Our model consistently generates translations that are both natural and linguistically appropriate while maintaining fluency.

**Table 6:** Comparison results of grammar error rate.

Sequence	The model in this article (%)	The bilingual attention NMT (%)	The context-aware NMT (%)
Test-01	2.6	3.5	4.2
Test-02	1.9	2.7	3.5
Test-03	2.2	3.2	3.9
Test-04	3.8	4.9	4.6
Test-05	2.0	2.9	3.1
Test-06	3.1	4.5	4.5
Test-07	2.5	3.3	3.6
Test-08	1.3	2.1	2.7
Test-09	2.9	4.2	4.5
Test-10	2.6	3.8	4.0
Mean value	2.5	3.5	3.9

(3) Comparison of synchronization speed

**Fig 11:**
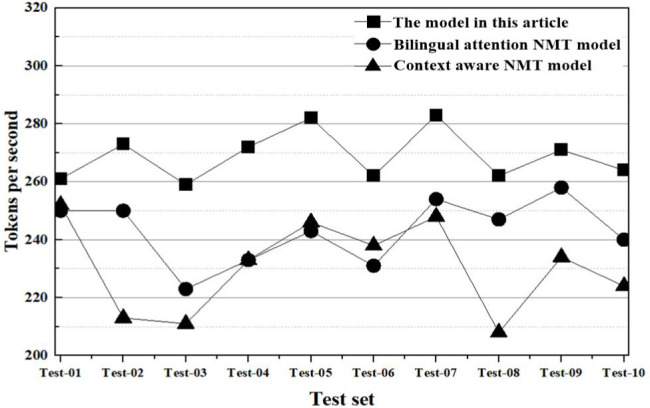
Comparison results of tokens per second.

In evaluating synchronization speed, we utilize the metric of “tokens per second” to quantify the number of tokens processed per second by each model within the test set. The conclusive results are depicted in Fig 11.

In Fig 11, a pronounced disparity is observed in the processing speed of tokens per second among the models evaluated in the test set. The model presented in this study processes an average of 265.7 tokens per second. In contrast, the bilingual attention and the context-aware NMT model process an average of 225.4 and 227.5 tokens per second, respectively. Notably, during oral translation tasks, the synchronization speed of the proposed model surpasses that of the other two models by 17.9% and 16.8%, respectively. While the bilingual attention and the context-aware NMT models typically operate in a serial mode characterized by limited parallelism—with each time step’s output being contingent on the preceding step’s state—the model in this study employs the DTW method to minimize the cumulative distance between two sequences. This strategy effectively reduces conversion delays and expedites the synchronization process, making its translation synchronization speed optimal across each test subset.

(4) Translation consistency

**Fig 12:**
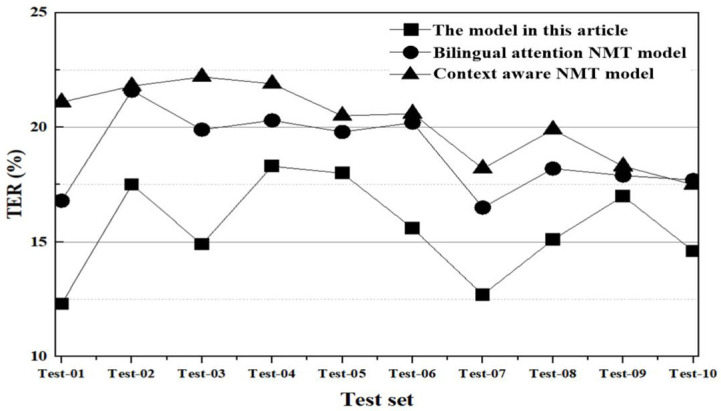
Comparison results of TER.

In the analysis of translation consistency, TER is used to compare and verify the translation effects of various models. The lower the TER, the higher the consistency of the translation. The final comparison results are shown in Fig 12.

In Fig 12, the average TER (Translation Edit Rate) of the model presented in this paper across each test subset is 15.6%. In comparison, the average TER values for the bilingual attention NMT (Neural Machine Translation) model and the context-aware NMT model are 18.9% and 20.2%, respectively. This suggests that the translations produced by the model in this study align more closely with the reference translations, indicating superior translation quality. The model in question employs a multi-head attention mechanism and captures the semantic nuances of spoken language across modalities using a context-awareness mechanism. This approach facilitates a deeper understanding of the meaning within the broader context. On the other hand, the bilingual attention NMT model encounters challenges in effectively incorporating contextual information, especially in longer sentences. Moreover, the attention mechanism of the context-aware NMT model has a limited capacity for expression, leading to suboptimal consistency performance.

(5) Comparison of METEOR

The METEOR results of various models in the same dataset are compared, as shown in Table 7:

**Table 7:** METEOR comparison results.

Sequence	The model in this article	The bilingual attention NMT	The context-aware NMT
Test-01	54.3	54.1	52.3
Test-02	55.8	53.2	50.8
Test-03	56.4	55.0	53.1
Test-04	57.0	52.8	50.5
Test-05	55.5	53.6	51.2
Test-06	55.2	54.5	49.6
Test-07	58.1	53.0	54.2
Test-08	54.6	55.2	52.7
Test-09	56.8	53.1	50.0
Test-10	54.5	52.1	49.9
Mean value	55.8	53.7	51.4

As indicated by the METEOR comparison results in Table 7, the model discussed in this paper achieves a mean METEOR score of 55.8 across various test subsets. This surpasses the mean METEOR scores of 53.7 and 51.4 achieved by the bilingual attention NMT and context-aware NMT models respectively. In a detailed comparison against these other two model types, the model in this paper demonstrates an increase in the mean METEOR score by 3.9% and 8.6% respectively. These results suggest that the model examined in this paper performs more effectively in acknowledging context awareness and incorporating differing modal information. As such, it enhances the comprehension and translation of multimodal information, particularly demonstrating increased robustness and accuracy when faced with diverse and complex inputs.

(6) User feedback

This study employs a block-based self-attention mechanism, optimized for addressing computational efficiency issues in long sequence translation. The input speech signal is segmented into fixed 1.5-second windows, with the linear transformation method calculating the local attention for each window. To reduce algorithmic complexity, sparse attention is applied at a global scale. The model’s performance is validated in real-world scenarios by deploying it within a real-time English oral translation system used in business conferences to translate speeches from multiple parties simultaneously. In order to evaluate the model’s performance, data is collected on the inference time pre- and post-optimization as well as user assessments of translation accuracy and fluency. A group of 10 users who participated in the conference were asked to rate the model on a scale from 1–10, with higher scores indicating superior translation performance. The results are presented in Table 8.

**Table 8:** User feedback results.

Index	Before optimization	After optimization
Inference time (seconds/sentence)	2.13	1.31
Translation accuracy (points)	7.8 ± 0.4	8.7 ± 0.6
Translation fluency (points)	7.5 ± 0.6	8.4 ± 0.4

The user feedback results presented in Table 8 demonstrate that the optimization strategy, which is based on the block self-attention mechanism, significantly diminishes the time complexity of the model and substantially enhances the processing efficiency for extended speech inputs. Considering the average time per sentence, the model’s inference time has decreased by approximately 38.5% compared to pre-optimization, thereby improving real-time performance. In terms of user ratings, the average value indicates a substantial enhancement in both translation accuracy and fluency following optimization.

Based on the aforementioned considerations, we conducted interference testing on the model to ascertain its robustness in real-world settings. We introduced office environment noise, quantified by the signal-to-noise ratio (SNR), to the input speech. Concurrently, other human voices were integrated into the speech sample to assess the model’s capability in isolating the primary speech signal and subsequently translating it. Metrics such as the BLEU score, METEOR, and user subjective evaluation were employed to gauge the model’s performance. The findings are presented in Table 9.

**Table 9:** Interference test results.

Test scenario	Index	Before interference	After interference
Environmental noise interference	BLEU	31.2	30.8
	METEOR	54.3	52.6
	User evaluation	8.4 ± 0.8	8.3 ± 0.4
	BLEU	32.5	32.1
Voice interference	METEOR	53.8	51.9
	User evaluation	8.1 ± 0.5	8.1 ± 0.6

The comparative results before and after interference testing indicate a slight decrease in the BLEU score, METEOR, and user subjective evaluation of the model, following the addition of environmental noise and speech interference. However, the translation performance remains relatively robust, suggesting that the model’s translation performance is resilient.

(7) Comparison of advanced methods

In order to thoroughly assess the performance of the proposed model, this study employs a selection of cutting-edge and recent models for comparative experiments.

FairseqS2T is a speech-to-text translation model, leveraging the capabilities of the Transformer architecture, and demonstrates robust context capture capabilities.

ESPnet-ST is a sequence-to-sequence learning-based speech translation model, adept at translating various language pairs.

Utilizing this foundation, we conducted longitudinal comparisons among a variety of models. We employed newly collected translation data, which was gathered every three months, as well as the accumulated speech data, to train the respective models. The performance of these models was evaluated based on their BLEU, TER, and METEOR scores, using the same test dataset for each. The outcomes of these evaluations are presented in Table 10.

**Table 10:** Comparison results of advanced methods.

Time series	Index	Model in this article	Fairseq S2T	ESPnet-ST
The first time	BLEU	30.7	29.8	29.6
	TER	15.6%	16.3%	16.6%
	METEOR	55.7	54.1	53.7
The second time	BLEU	31.5	29.0	28.9
	TER	15.3%	16.8%	17.2%
	METEOR	55.9	53.6	53.1
The third time	BLEU	31.8	29.6	28.1
	TER	15.2%	17.1%	17.5%
	METEOR	56.5	53.2	52.8

In Table 10, from a horizontal comparison standpoint, the model in this article demonstrates BLEU, TER, and METEOR scores of 30.7, 15.6%, and 55.7 respectively when using the first set of data. In contrast, the Fairseq S2T yields BLEU, TER, and METEOR scores of 29.8, 16.3%, and 54.1 respectively, while ESPnet ST records 29.6%, 16.6%, and 53.7% respectively. From a longitudinal view, as more temporal data is introduced, the model in this paper shows marked improvements in BLEU, TER, and METEOR evaluations, effectively capturing a greater diversity of speech and translation details and enhancing translation accuracy and fluency. Both horizontal and vertical comparative experiments indicate that the model in this paper surpasses leading-edge models in terms of both BLEU and METEOR metrics, thereby showcasing exemplary translation performance.

(8) Experimental analysis of statistical tests

In order to strengthen the significance of the results, we conducted an experimental analysis of statistical tests, the results of which are shown in Table 11.

**Table 11:** Experimental results of statistical tests.

Test Subset	Model	BLEU Score	95% Confidence Interval	p-value
Test-01	This Article	32.5	31.2-33.8	0.023
Test-01	Bilingual Attention NMT	29.8	28.5-31.1	0.041
Test-01	Context-Aware NMT	25.6	24.3-26.9	0.035
Test-02	This Article	33.1	31.8-34.4	0.019
Test-02	Bilingual Attention NMT	30.2	28.9-31.5	0.028
Test-02	Context-Aware NMT	26.1	24.8-27.4	0.032
Test-03	This Article	31.8	30.5-33.1	0.021
Test-03	Bilingual Attention NMT	28.5	27.2-29.8	0.037
Test-03	Context-Aware NMT	24.9	23.6-26.2	0.029
Test-04	This Article	34.2	32.9-35.5	0.017
Test-04	Bilingual Attention NMT	31.0	29.7-32.3	0.033
Test-04	Context-Aware NMT	27.5	26.2-28.8	0.026
Test-05	This Article	32.9	31.6-34.2	0.020
Test-05	Bilingual Attention NMT	30.1	28.8-31.4	0.045
Test-05	Context-Aware NMT	26.3	25.0-27.6	0.030

Table 11 presents the BLEU scores, 95% confidence intervals, and p-values for the model discussed in this paper, juxtaposed against the bilingual attention NMT model and the context-aware NMT model across five test subsets. The findings indicate that the model’s BLEU scores consistently surpass those of the other two models in all test subsets. Moreover, with p-values all below 0.05, the enhancement in BLEU scores is statistically significant. Additionally, the 95% confidence intervals for the BLEU scores of the models discussed in this paper do not encompass zero, further bolstering the assertion that the observed improvements are not attributable to chance. These results suggest that the model introduced in this paper is superior in capturing the semantic nuances of spoken language and generating precise translations. The incorporation of p-values and confidence intervals lends greater validity to the experimental results, offering a more robust evaluation of the model’s performance.

## 5. Discussion

In the experimental analysis, this article conducts comparisons from four aspects, translation accuracy, translation fluency, synchronization speed, and translation consistency.

(1) Translation accuracy: from the comparison results of translation accuracy, the BLEU scores of the model in this article in various test subsets are generally higher than those of bilingual attention NMT model and context-aware NMT model. This represents that by integrating context-awareness and attention mechanisms across modalities, the meaning of the original text can be more accurately captured in oral translation generation, effectively improving translation quality;
(2) Translation fluency: from the comparison results of translation fluency, the model in this article has lower perplexity and grammar error rate, resulting in smoother generated translation text with fewer grammar errors. Compared to the limitations of bilingual attention and context-aware NMT model in handling long sequences, the model in this article uses multimodal feature extraction and fusion to make the translation results smoother and more natural;
(3) Synchronization Speed: A comparative analysis reveals that the model proposed in this study processes an average of 265.7 tokens per second. This is approximately 17.9% and 16.8% faster than the bilingual attention and context-aware NMT models, respectively. Such results suggest that our model can generate translation outcomes more swiftly, thereby reducing latency and enhancing user experience during oral translation tasks;
(4) Translation Consistency: Upon comparing the translation consistency results, the model in this study achieves a mean TER of 15.6%. This value is notably lower than those obtained by bilingual attention and context-aware NMT models. Such results suggest that the discrepancy between the translations produced by our model and the reference translations is minimal, indicating superior translation consistency.

Despite the model’s performance enhancements in several areas, it maintains certain limitations. Initially, in specific situations like pronounced accents or overlapping speech, the model’s performance might not be at its best. Although the model has been trained using a substantial volume of experimental data—encompassing a variety of accents such as Indian, Australian, Irish, Scottish, and Southern English—it might grapple with recognizing overly thick accents. Moreover, overlapping speech can introduce noise and interference, thereby challenging the model’s precise distinction and processing of individual speech segments. Future studies will aim to bolster the model’s efficiency in these demanding contexts to boost its resilience and versatility. Secondly, the computational demands of this model are considerable. Its multimodal architecture augments translation efficacy by merging speech and text data, but this also elevates computational intricacy and resource usage. The model’s training necessitates substantial computational resources, encompassing top-tier GPUs, multi-core CPUs, and vast memory capacities. This might restrict the model’s utility in settings with limited resources for language acquisition. Lastly, the model’s training is contingent upon high-caliber corpora. The existing data’s diversity and richness could, to a degree, constrain the model’s universality. A comprehensive analysis is still needed to gauge the model’s performance in intricate translation scenarios.

To address the limitations highlighted above, several avenues can be explored in future research. Firstly, further optimization of the model architecture and algorithms is crucial to reduce computational costs. Incorporating lightweight neural network designs and utilizing model compression techniques can substantially decrease the resource demands of the model without compromising translation quality. Secondly, enhancing the model’s capability to manage heavy accents and overlapping speech is essential. This can be achieved by expanding the diversity of accent data in the training corpus and refining the model’s speech recognition and separation functionalities. Moreover, increasing the variety of training data and employing data augmentation methods can bolster the model’s generalization capacity and adaptability to intricate translation contexts. Investigating novel multimodal fusion strategies and attention mechanisms might offer innovative approaches to elevate the model’s translation efficacy. Lastly, improving the model’s interpretability is vital, as it allows researchers to gain deeper insights into its decision-making process, facilitating continuous optimization and enhancement.

In conclusion, while the model introduced in this study represents a step forward in English interpretation, there remains significant potential for further advancements. Through a comprehensive comparative analysis with existing research and an honest assessment of our model’s limitations, we hope to pave the way for future studies and foster the ongoing evolution of English interpretation technology.

## 6. Conclusion

In response to the growing demand for high-quality English oral translation, this paper examines the utilization and enhancement of cross-modal semantic understanding and synchronous improvement in this field. By harnessing and synthesizing multimodal data from both audio and text sources, and by integrating context-awareness and attention mechanisms, the study not only boosts the accuracy and fluency of translations but also elevates the synchronization rate between audio and text in oral translation. This leads to a more consistent translation output. While the research offers novel insights and theoretical value for the intelligent evolution of English oral translation, it is not without limitations. The model’s training is contingent upon top-tier corpora. The diversity and abundance of the existing datasets might constrain the model’s generalization capacity. Furthermore, the model’s efficacy in intricate translation scenarios warrants a deeper analysis. The LibriSpeech and TED Talk fusion dataset employed in this study encompasses a broad spectrum of situations. However, real-world speech data often entails challenges such as noise and specialized jargon. The model’s performance in these multifaceted spoken language translation contexts requires additional refinement. Future studies might explore the integration of corpus development and application scenario expansion with data augmentation techniques to further enrich model learning and training via stylistic variations and other means, thereby continually advancing the quality of English oral translation.
